# Exploring human-genome gut-microbiome interaction in Parkinson’s disease

**DOI:** 10.1038/s41531-021-00218-2

**Published:** 2021-08-18

**Authors:** Zachary D. Wallen, William J. Stone, Stewart A. Factor, Eric Molho, Cyrus P. Zabetian, David G. Standaert, Haydeh Payami

**Affiliations:** 1 Department of Neurology, University of Alabama at Birmingham, Birmingham, AL USA; 2 Department of Neurology, Emory University School of Medicine, Atlanta, GA USA; 3 Department of Neurology, Albany Medical College, Albany, NY USA; 4 VA Puget Sound Health Care System and Department of Neurology, University of Washington, Seattle, WA USA

**Keywords:** Risk factors, Parkinson's disease, Genotype, Genetic association study

## Abstract

The causes of complex diseases remain an enigma despite decades of epidemiologic research on environmental risks and genome-wide studies that have uncovered tens or hundreds of susceptibility loci for each disease. We hypothesize that the microbiome is the missing link. Genetic studies have shown that overexpression of alpha-synuclein, a key pathological protein in Parkinson’s disease (PD), can cause familial PD and variants at alpha-synuclein locus confer risk of idiopathic PD. Recently, dysbiosis of gut microbiome in PD was identified: altered abundances of three microbial clusters were found, one of which was composed of opportunistic pathogens. Using two large datasets, we found evidence that the overabundance of opportunistic pathogens in PD gut is influenced by the host genotype at the alpha-synuclein locus, and that the variants responsible modulate alpha-synuclein expression. Results put forth testable hypotheses on the role of gut microbiome in the pathogenesis of PD, the incomplete penetrance of PD susceptibility genes, and potential triggers of pathology in the gut.

## Introduction

Parkinson’s disease (PD) affects over 6 million people worldwide, having doubled in one decade, and continues to rapidly increase in prevalence with the aging of the world population^1^. PD is a progressive degenerative disease which affects the brain, the peripheral nervous system, and the gastrointestinal tract, causing progressive, debilitating movement disorders, gastrointestinal and autonomic dysfunction, sleep disorders, and cognitive impairment. Currently, there is no prevention, cure, or treatment known to slow the progression of the disease.

Like other common late-onset disorders, PD has Mendelian forms caused by rare mutations, but the vast majority of cases remain idiopathic. Both genetic and environmental risk factors have been identified^2–4^, but none have large enough effect sizes individually or in combination to fully encapsulate disease risk^5–8^. The triggers that initiate the onset of PD pathology are unknown.

There is a connection between PD and the gastrointestinal tract^9,10^ and the gut microbiome^11^. The gut microbiome is a relatively new and increasingly active area of research in human disease^12–14^. Studies on PD have consistently found altered gut microbiome, with depletion of short-chain fatty acid (SCFA) producing bacteria, and enrichment of *Lactobacillus* and *Bifidobacterium*^11,15,16^. Most studies to date have been modest in size, and therefore have examined mostly common microorganisms. We recently reported a microbiome-wide association study in PD, using two large datasets and internal replication, which enabled investigation of less common taxa not reported before^11^. In these datasets, reduced SCFA-producing bacteria and elevated *Lactobacillus* and *Bifidobacterium* were robustly confirmed. In addition, a significant increase was detected in the relative abundance of a poly-microbial cluster of opportunistic pathogens, including *Corynebacterium_1* (*C. amycolatum, C. lactis*)*, Porphyromonas* (*P. asaccharolytica, P. bennonis, P. somerae, P. uenonis*), and *Prevotella* (*P. bivia, P. buccalis, P. disiens, P. timonensis*). These are commensal bacteria with normally low abundance in the gut; in fact, *Corynebacterium* is commensal to skin microbiome not the gut. They are referred to as opportunistic pathogens in the literature (as opposed to pathobionts) because they are not prevalent native members of the gut, rather they are known to be able to cause infections in any part of the body if they gain access to a sterile site through wounds, surgery or permeable membranes and are allowed to grow due to a compromised immune system (literature reviewed in ref. ^11^).

Overabundance of opportunistic pathogens in PD gut was of interest because it harks back to the hypothesis advanced by Professor Heiko Braak which proposes that in non-familial forms of PD, the disease is triggered by an unknown pathogen in the gut and spreads to the brain^17,18^. Braak’s hypothesis was based on pathological studies of postmortem human brain, stained using antibodies to alpha-synuclein. Misfolded alpha-synuclein, the pathologic hallmark of PD, has been seen to form in enteric neurons early in disease^19–21^, and has been shown to propagate in a prion-like manner from the gut to the brain in animal models^22^. The gene that encodes alpha-synuclein is *SNCA*. *SNCA* gene multiplication results in drastic overexpression of alpha-synuclein and causes Mendelian autosomal dominant PD. Variants in the *SNCA* region are associated with risk of idiopathic PD^23^, and are expression quantitative trait loci (eQTL) associated with expression levels of *SNCA*^24–26^. Increased alpha-synuclein expression has been noted with infections unrelated to PD^27,28^. We hypothesized that if opportunistic pathogens are involved in disease pathogenesis, there might be an interaction between genetic variants in the *SNCA* region and dysbiosis of the gut in PD.

## Results

### Overview of analyses

The two case-control cohorts used here are those previously employed by Wallen et al. to characterize the PD gut microbiome^11^. Here, we generated and added genotype data to investigate interactions. The sample size for the present analysis was 199 PD and 117 controls in dataset 1, and 312 PD and 174 controls in dataset 2. All samples had complete genotypes, 16S microbiome data, and metadata (Supplementary Table 1).

We defined the boundaries of the *SNCA* region such that it would encompass known *cis*-eQTLs for *SNCA*. Using GTEx eQTL database, we defined the boundaries as ch4:88.9 Mb, downstream of 3′ *SNCA*, and ch4:90.6 Mb, upstream of 5′ *SNCA*. In our genome-wide genotype data (see Methods), we had 2,627 single nucleotide polymorphisms (SNPs) that mapped to this region, had minor allele frequency (MAF) >0.1, imputation quality score *r*^2^ >0.8, and were in common between the two datasets being studied here.

The taxa examined were grouped and analyzed at genus/subgenus/clade level as *Corynebacterium_1* (*C. amycolatum, C. Lactis*)*, Porphyromonas* (*P. asaccharolytica, P. bennonis, P. somerae, P. uenonis*), and *Prevotella* (*P. bivia, P. buccalis, P. disiens, P. timonensis*). For simplicity, we will refer to the three microbial groups as taxa. As we have previously shown, the abundances of these taxa are elevated in PD vs. control. These findings were replicated in the two datasets (Table 1), verified by two statistical methods, robust to covariate adjustment (over 40 variables investigated), and yielded no evidence of being the result of PD medications or disease duration^11^.

**Table 1:** Stratified analyses suggest increased abundance of opportunistic pathogens in PD gut microbiome is dependent on the host genotype.

	N PD N Control	OR [95%CI]	*P*	N PD N Control	OR [95%CI]	*P*	N PD N Control	OR [95%CI]	*P*	N PD N Control	OR [95%CI]	*P*
(a) *Corynebacterium_1*	All subjects			rs356229_TT			rs356229_TC			rs356229_CC		
Dataset 1	199 117	1.5 [1.1–2.1]	0.02	64 53	1.0 [0.6–1.8]	0.90	90 48	1.7 [1.1–2.7]	0.03	45 16	2.6 [0.9–7.2]	0.08
Dataset 2	312 174	1.7 [1.0–2.9]	0.05	107 66	0.8 [0.3–2.1]	0.70	150 80	2.6 [1.2–5.4]	0.01	55 28	2.3 [0.6–8.5]	0.21
Meta-analysis	511 291	1.6 [1.2–2.1]	3E−3	171 119	1.0 [0.6–1.6]	0.92	240 128	1.9 [1.3–2.8]	1E−3	100 44	2.5 [1.1–5.6]	0.03

(b) *Porphyromonas*	All subjects			rs10029694_GG			rs10029694_GC			rs10029694_CC		
Dataset 1	199 117	2.1 [1.4–3.2]	4E−4	154 94	1.5 [1.0–2.4]	0.06	43 22	5.2 [2.0–13.8]	1E−3	2 1	64.3 [0.6–7155.8]	0.33
Dataset 2	312 174	1.9 [1.2–3.1]	7E−3	251 142	1.6 [1.0–2.7]	0.06	57 28	3.4 [0.9–12.6]	0.07	4 4	48.1 [1.1–2125.6]	0.12
Meta-analysis	511 291	2.0 [1.5–2.8]	7E−6	405 236	1.6 [1.1–2.2]	7E−3	100 50	4.5 [2.1–9.8]	2E−4	6 5	53.9 [2.8–1032.6]	8E−3

(c) *Prevotella*	All subjects			rs6856813_TT			rs6856813_TC			rs6856813_CC		
Dataset 1	199 117	2.1 [1.4–3.2]	9E−4	72 48	2.6 [1.4–4.7]	3E−3	91 57	1.6 [0.9–3.0]	0.12	36 12	1.8 [0.4–8.7]	0.49
Dataset 2	312 174	2.4 [1.5–3.8]	2E−4	119 69	5.6 [2.7–11.8]	1E−5	143 77	1.9 [1.0–3.7]	0.05	50 28	0.8 [0.3–2.1]	0.60
Meta-analysis	511 291	2.2 [1.6–3.0]	4E−7	191 117	3.5 [2.2–5.7]	2E−7	234 134	1.8 [1.1–2.8]	0.01	86 40	1.0 [0.4–2.3]	0.95

Testing the abundances of three taxa in PD vs. control. (a) *Corynebacterium_1*, (b) *Porphyromonas*, and (c) *Prevotella* (as defined by SILVA taxonomic database) were elevated in PD gut microbiome, as reported previously^11^, and shown here in the first panel (all subjects). The differential abundance was then tested within each genotype of the interacting SNP. Results are consistent across the two datasets and summarized by meta-analysis, showing differential abundance of opportunistic pathogens in PD is genotype-dependent. PD: number of subjects with Parkinson’s disease; Cont: number of control subjects; OR [95%CI]: odds ratio and 95% confidence interval estimating the fold-change in clr-transformed taxa abundance in PD vs. control; *P*: statistical significance. Clr-transformed abundance of each taxon was tested in PD vs controls using linear regression adjusted for sex and age. Formal test of heterogeneity across datasets revealed no heterogeneity, thus a fixed-model was used for meta-analysis.

**Fig. 1:**
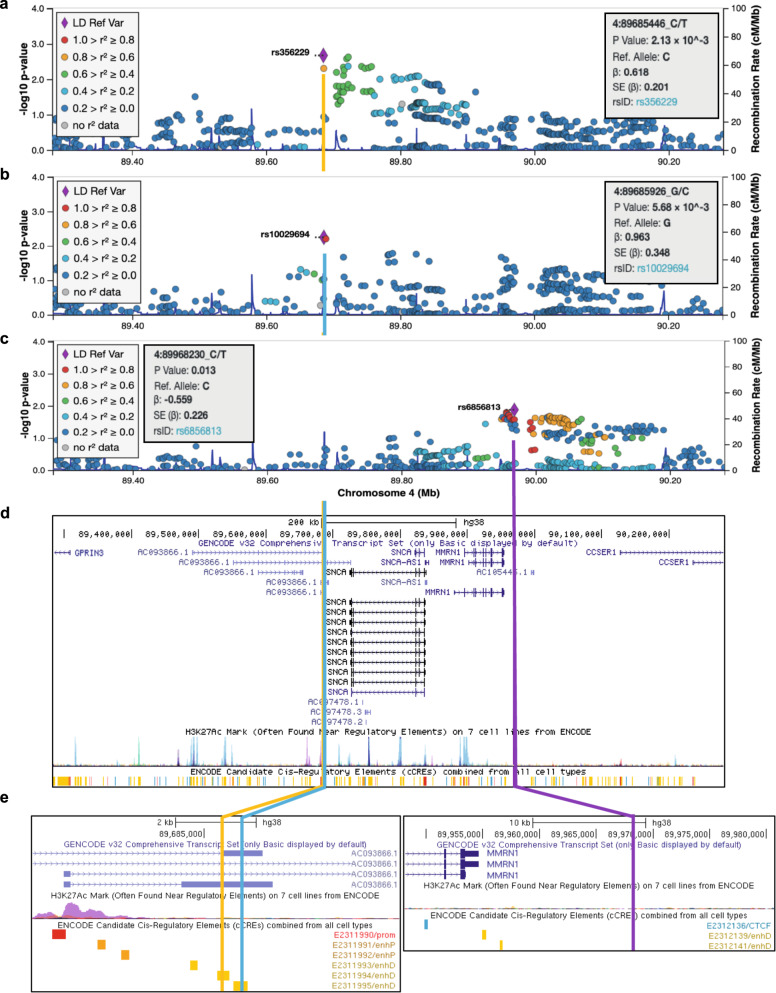
Genetic map of candidate interacting SNPs. SNPs in *SNCA* region (chromosome 4: 88.9–90.6 Mb) were tested for interaction on the association of three taxa with PD. Results are shown in LocusZoom, where each SNP is plotted according to its base pair position and meta-analysis −log10(*P* value) for interaction for the three taxa: (**a**) *Corynebacterium_1*, (**b**) *Porphyromonas*, and (**c**) *Prevotella*. The SNP with the highest significance is shown as a purple diamond, and was chosen as candidate interacting SNP for stratified analysis (Table 1). (**d**) UCSC Genome Browser shows the interacting SNPs for *Corynebacterium_1* and *Porphyromonas* map to 3′ *SNCA* in a lncRNA that overlaps with and are antisense to *SNCA*. The interacting SNP for *Prevotella* is distal at 5′ of *SNCA* and *MMRN1*. (**e**) The interacting SNPs for *Corynebacterium_1* and *Porphyromonas*, while only 450 base pair apart, are not in LD (*R*^2^ = 0) and map to adjacent regulatory sequences shown in yellow bars. The interacting SNP for *Prevotella* does not map to any known functional sequence. All three SNPs are eQTLs for *SNCA* and lncRNA genes *SNCA-AS1, RP11-115D19.1* (*AC093866.1*), and *RP11-115D19.2* (*AC097478.2*) which are associated with expression of *SNCA* (Table 2). LD: linkage disequilibrium; Mb: Megabase; *P* value: *P* value from meta-analysis; β: beta coefficient of interaction from meta-analysis; SE: standard error; rsID: reference SNP ID for the marked SNP.

**Fig. 2:**
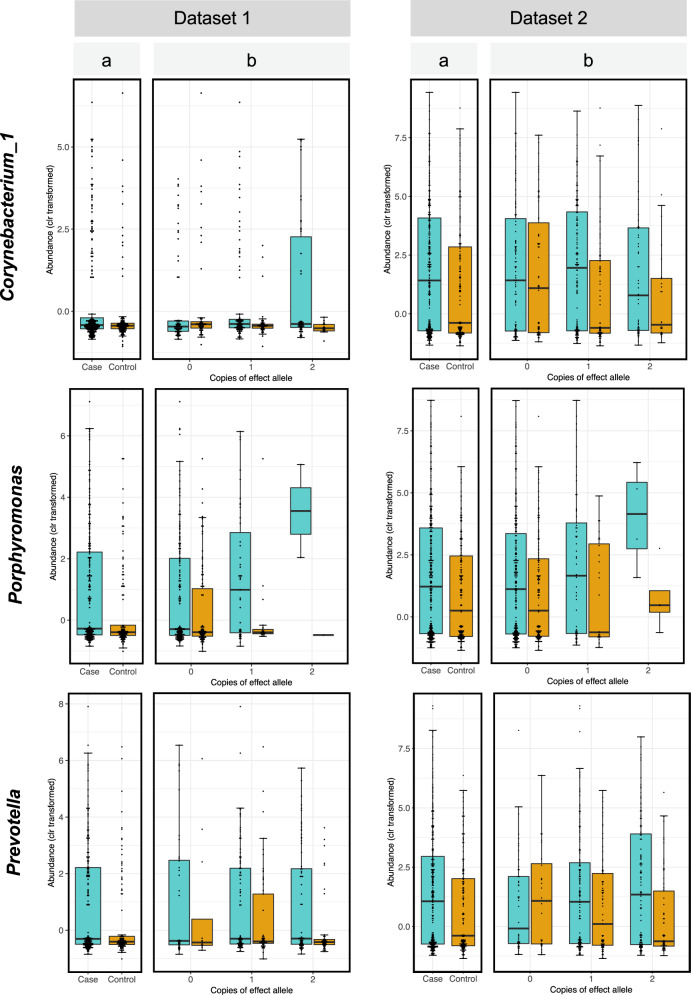
Differential abundance of opportunistic pathogens. Clr-transformed abundances of each taxon are plotted for PD cases (blue) and controls (orange) for all subjects irrespective of genotype (panel a) and stratified for the three genotypes of the interacting SNP (panel b). The bottom, middle, and top boundaries of each box represent the first, second (median), and third quartiles of the clr-transformed abundances. Lines extending from the top and bottom of boxes show 1.5 times the interquartile range. Points extending above or below the horizontal caps of the top and bottom lines of each box are outliers. The two datasets show the same pattern of interaction where the difference between PD and controls in the abundances of each taxon becomes larger with increasing number of the effect allele. Dataset 2 has higher resolution than dataset 1 (particularly for *Corynebacterium_1* which is rare) because it had 10x greater sequencing depth.

The analysis of interaction was structured as follows. First, we screened for statistical interaction between 2,627 SNP genotypes in the *SNCA* region, case-control status, and centered log-ratio (clr) transformed abundance of each taxon and selected the SNP with the highest statistical significance as the candidate interacting SNP (Fig. 1a–c). We then tested the association of each taxon with case-control status after stratifying the subjects by the interacting SNP genotype. The effect of SNP on the PD-taxa association was tested statistically (Table 1) and illustrated graphically (Fig. 2, Supplementary Fig. 1). We tested the association of interacting SNPs with PD in the present dataset and in prior GWAS (Table 2). This test was conducted because interaction can exist with or without a main effect of SNP on disease risk. We then conducted in silico functional analysis of the interacting SNPs (Table 2, Fig. 1d, e). All analyses were performed in two datasets, followed by meta-analysis.

**Table 2:** Characteristics of the interacting variants at *SNCA* locus.

PD-associated taxa	Interacting SNP at *SNCA*	Interaction *P*	Effect allele	Effect allele freq.	a. Association with PD		b. Association with gene expression			
					Present study OR (*P*)	GWAS OR (*P*)	Gene	eQTL *P*	Tissue studied	Source
*Corynebacterium_1*	rs356229_T/C	2E−3	C	0.4	1.3 (0.04)	1.3 (3E−42)	*SNCA*	1E−13	Whole blood	eQTLGen
							*SNCA*	9E−5	Esophagus mucosa	GTEx
							*SNCA-AS1*	2E−7	Pituitary	GTEx
							*RP11-115D19.1*	3E−14	Skin	GTEx
							*MMRN1*	5E−5	Spleen	GTEx
							*MMRN1*	4E−9	Whole Blood	eQTLGen
*Porphyromonas*	rs10029694_G/C	6E−3	C	0.1	1.1 (0.62)	1.1 (2E−14)	*RP11-115D19.1*	1E−5	Skin	GTEx
							*RP11-115D19.2*	7E−6	Skin	GTEx
*Prevotella*	rs6856813_T/C	0.01	T	0.6	0.9 (0.43)	—	*SNCA*	3E−49	Whole blood	eQTLGen
							*SNCA*	2E−5	Artery-Tibial	GTEx
							*SNCA*	1E−4	Artery-Aorta	GTEx
							*MMRN1*	3E−11	Whole blood	eQTLGen

Test of statistical interaction nominated three different and independent single nucleotide variants (SNPs) at *SNCA* region as modifiers of the relative increase of three opportunistic pathogens in PD gut microbiome. (a) Two of the variants were detected in prior GWAS as being directly associated with PD. Association of rs356229_T/C with PD was detected in a GWAS meta-analysis conducted in 2014 with ~19,000 PD cases and ~100,000 controls^23^. Association of rs10029694 with PD was detected in a larger GWAS meta-analysis conducted in 2019 with 37,688 PD cases and 1.4 million controls^3^. (b) All three SNPs are expression quantitative loci (eQTL) for *SNCA*, lncRNA antisense to *SNCA* known to regulate *SNCA* expression (*SNCA-AS1*, *RP11-115D19.1)*, lncRNA *RP11-115D19.2* which is embedded in and antisense to *SNCA,* and *MMRN1*, a protein coding gene (mutimerin 1) upstream of 5′ *SNCA* which is often multiplicated along with *SNCA* multiplication in familial PD. Data were obtained from eQTL databases GTEx and eQTLGen. Important to note that the names of genera are not standardized across reference databases and caution should be exercised when comparing results from different studies; these genera were defined using SILVA reference database. Effect allele: variant of interacting SNP that is associated with increased differential abundance of the taxon in PD vs. controls. pdgene.org: catalogue of PD-associated genes. *RP11-115D19.1* is denoted as *AC093866.1* in Fig. 1, *RP11-115D19.2* is denoted as *AC097478.2* in Fig. 1.

#### Corynebacterium_1

The candidate interacting SNP for *Corynebacterium_1* was rs356229 (interaction *P* = 2E−3; Fig. 1a). This SNP is located 3′ of *SNCA* (Fig. 1a, d). The two alleles are rs356229_T (allele frequency = 0.6) and rs356229_C (frequency = 0.4), and was imputed with imputation quality score of 0.96 in dataset 1 and 0.99 in dataset 2.

If we do not consider genotype, *Corynebacterium_1* abundance is significantly elevated in PD (OR = 1.6, *P* = 3E−3). However, when data are stratified by genotype, there is no association between *Corynebacterium_1* and PD among individuals with rs356229_TT genotype, who comprised 36% of the study (OR = 1.0, *P* = 0.92). The association of *Corynebactreium_1* with PD was dependent on the presence of the rs356229_C allele. The abundance of *Corynebactreium_1* was nearly 2-fold higher in PD than controls in heterozygous rs356229_CT (OR = 1.9, *P* = 1E−3), and 2.5-fold higher in the homozygous rs356229_CC individuals (OR = 2.5, *P* = 0.03). These results can be found in Table 1a, and data underlying these results are visualized in Fig. 2.

The *Corynebacterium_1* interacting SNP has been previously identified in PD GWAS meta-analysis, with the rs356229_C allele associated with increased PD risk (OR = 1.3, *P* = 3E−42 with *N*>100,000 samples^23^). We also detected an association between rs356229_C and PD in the present dataset (OR = 1.3, *P* = 0.04 with *N* = 802 samples; Table 2). That we estimated an effect size identical to GWAS, despite the enormous disparity in the sample size and power, speaks to the robustness of the data. The evidence for interaction does not stem from the association of SNP with PD (see “Discussion”). Interestingly, the association of rs356229_C with risk of PD varied by the increasing abundance of *Corynebacterium*_1 from no association in the 1st or 2nd quartile (OR = 0.9, *P* = 0.5; OR = 1.1, *P* = 0.8) to an emerging and then strong association in the 3rd and 4th quartiles (OR = 1.5, *P* = 0.3 and OR = 2.2, *P* = 5E−3).

The rs356229 SNP maps to a distal regulatory element at 3′ of *SNCA* (Fig. 1d, e) and is an eQTL for *SNCA* (Table 2). Data were obtained by eQTL GWAS conducted in whole blood (eQTLGen.org) and in esophagus mucosa (GTExportal.org). The rs356229_C allele is associated with increased expression of *SNCA* in blood (eQTL *P* = 1E−13) and in esophagus mucosa (eQTL *P* = 9E−5). According to GTEx, rs356229 is also an eQTL for *SNCA-AS1* (eQTL *P* = 2E−7) and *RP11-115D19.1* (eQTL *P* = 3E−14). *SNCA-AS1* and *RP11-115D19.1* overlap with *SNCA* and encode long non-coding RNA (lncRNA) that are antisense to *SNCA* (Fig. 1d) and have been implicated in the regulation of *SNCA* expression^29–31^.

#### Porphyromonas

The candidate interacting SNP for *Porphyromonas* was rs10029694 (interaction *P* = 6E−3; Fig. 1b). The SNP maps to 3′ of *SNCA* (Fig. 1b, d). The two alleles are rs10029694_G (frequency = 0.9) and rs10029694_C (frequency = 0.1), and was imputed with imputation quality score 0.99 in dataset 1 and 0.92 in dataset 2.

The interacting SNPs for *Porphyromonas* (rs10029694) and *Corynebacterium_1* (rs356229) map very close to each other, only 480 base pairs apart, but they are not in linkage disequilibrium (LD): *D*′<0.01, *R*^2^ = 0.

*Porphyromonas* was elevated in PD irrespective of rs10029694_G/C genotype (OR = 2.0, *P* = 7E−6), and in every genotype, but the statistical interaction implied difference across genotypes. Shown in stratified analysis (Table 1b, Fig. 2), the rs10029694_GG genotype had a nearly two-fold higher abundance of *Porphyromonas* in PD vs. controls (OR = 1.6, *P* = 7E−3), rs10029694_GC had nearly five-fold difference (OR = 4.5, *P* = 2E−4) and rs10029694_CC had approximately 54-times higher abundance of *Porphyromonas* in PD than in controls (OR = 53.9, *P* = 8E−3). Note however that there were only 11 individuals with rs10029694_CC genotype. Although the statistical methods were carefully chosen to be robust to small sample size, and the *P* value is quite significant despite the sample size, the fact remains that the OR = 54 was generated on only 11 people. If we collapse the rare rs10029694_CC genotype with rs10029694_CG, we have 161 individuals (20% of subjects) with at least one copy of rs10029694_C allele, and we get a more conservative estimate of OR = 5.1 (*P* = 2E−5) for association of *Porphyromonas* with PD in people with one or two copies of rs10029694_C.

The *Porphyromonas* interacting SNP is also associated with PD risk. The association was detected in the latest GWAS which had 37,688 PD cases and 1.4 million controls (OR = 1.1, *P* = 2E−14)^3^. We detected the same effect size (OR = 1.1) but it did not reach significance (*P* = 0.6) (Table 2). As would be expected from the interaction, the frequency of the effect allele rs10029694_C in PD vs. control rose with increasing abundance of *Porphyromonas*, yielding OR = 0.6 (*P* = 0.3) for 1st quartile and increasing up to OR = 2.2 (*P* = 0.08) for the 4th quartile.

The rs10029694 SNP maps to a distal regulatory element at 3′ of *SNCA*, adjacent to another regulatory element where rs356229, the interacting SNP for *Corynebacterium_1* resides (Fig. 1d, e). The rs10029694 SNP is an eQTL for two lncRNA that are antisense to *SNCA*: *RP11-115D19.1* (eQTL *P* = 1E−5) and *RP11-115D19.2* (eQTL *P* = 7E−6) (Table 2). *RP11-115D19.1* overlaps with 3′ of *SNCA; RP11-115D19.2* is within *SNCA*. We did not find direct evidence for rs10029694 being an eQTL for *SNCA*. However, *RP11-115D19.1* and *RP11-115D19.2* are antisense to *SNCA* which based on current knowledge on function of antisense lncRNA would be presumed to be regulatory for *SNCA*^30,31^, and *RP11-115D19.1* has been directly shown to regulate *SNCA* expression^29^.

#### Prevotella

The candidate interacting SNP for *Prevotella* was rs6856813 (interaction *P* = 0.01; Fig. 1c). The SNP is ~100 kb upstream at 5′ of *SNCA* (Fig. 1c, d). The two alleles are rs6856813_T (frequency = 0.6) and rs6856813_C (frequency = 0.4), and was imputed with imputation quality score 0.98 in dataset 1 and 0.84 in dataset 2. The *Prevotella* interacting SNP is 300Kb away from and not in LD with the interacting SNPs of *Corynebacterium_1* (rs356229, *D*′ = 0.2, *R*^2^ = 0.04) or *Porphyromonas* (rs10029694, *D*′ = 0.36, *R*^2^ = 0.01).

*Prevotella* was elevated two-fold in PD vs. controls (OR = 2.2, *P* = 4E−7). Genotype-specific results suggest rs6856813_TT had the greatest differential abundance in PD vs. control (OR = 3.5, *P* = 2E−7), followed by rs6856813_TC (OR = 1.8, *P* = 0.01), and no difference in rs6856813_CC genotype (OR = 1.0, *P* = 0.95) (Table 1c, Fig. 2).

There is no documented evidence for a direct association between rs6856813_C/T and PD in this study (OR = 0.9, *P* = 0.4) or in PD GWAS to date (Table 2). There is a statistically non-significant trend of increasing frequency of rs6856813_T allele with increasing abundance of *Prevotella* in PD, yielding OR = 0.8 in 1st quartile and increasing to OR = 1.5 in 4th quartile, consistent with the presence of interaction.

Although rs6856813 is ~100 kb upstream of S*NCA* and does not map to a known regulatory sequence (Fig. 1d, e), it is a strong eQTL for *SNCA*: the rs6856813_T allele, which is the effect allele for interaction with *Prevotella*, is associated with increased *SNCA* expression in blood (eQTL *P* = 3E−49) and in arteries (eQTL *P* = 2E−5) (Table 2).

## Discussion

Numerous studies have been performed on the association of genetic variants with PD and separately of gut microbiome and PD, but none to our knowledge has explored the interaction between the two. Here we have used a candidate taxa, candidate gene strategy: we used prior knowledge of the association of PD with elevated abundances of certain opportunistic pathogens in the gut^11^ and searched for genetic modifiers of these associations in the *SNCA* gene region^23^. Through statistical interaction tests we identified specific variants in the *SNCA* region as candidate interacting variants and through genotype-stratified analyses we found evidence suggesting that the increases in the relative abundance of opportunistic pathogens in PD gut are modulated by host genotype.

Statistical interaction tests provide a means to investigate if the association of one factor with the trait is influenced by a second factor. Here, we tested if the association of three opportunistic pathogens with PD (organisms with higher relative abundance in PD cases than similarly aged controls) is dependent on genetic variations in or around *SNCA*. Interaction studies require much larger sample sizes and power than association studies; the *P* values for interaction seldom achieve significance, and when they do, they are far less significant than the *P* values for a similarly sized one-factor association study. To that end, a major limitation of this study was the sample size. The raw *P* values from interaction tests were significant but did not pass multiple testing correction. While the test of interaction in itself is not a powerful statistical means to detect modifiers, it is an unbiased screen to narrow a large region down to a few potential candidates that can be further interrogated individually in stratified analysis. We nominate rs356229_T/C and rs10029694_G/C as potential modifiers for the association of *Coynebacterium_1* and *Porphyromonas* with PD based on the following evidence: (a) stratified analysis, on two datasets, showed similar and statistically significant differences in taxa abundance by genotype, (b) the SNPs are eQTL affecting *SNCA* expression, and (c) both SNPs have been shown in GWAS to be independently associated with PD. The evidence for interaction did not arise from and is independent of the direct association of the SNPs with PD. This can be seen in Table 1, where the test is between taxa and PD; SNP is not in the test, it is only used to divide the samples by genotype, which showed varying association between the taxon and PD as a function of genotypes in both datasets. In fact, the present dataset had marginal evidence for direct association of PD with rs356229_T/C (OR = 1.3, *P* = 0.04), and was not significant for rs10029694_G/C (OR = 1.1, *P* = 0.6). The evidence for direct associations with PD come from the 2014 GWAS meta-analyses which detected association of rs356229_T/C with PD at OR = 1.3, *P* = 3E−42 with *N* > 100,000 cases and controls^23^, and the 2019 GWAS met-analysis which detected association of rs10029694_G/C with PD at OR = 1.1 and *P* = 2E−14 with *N* > 1.4 million cases and controls^3^. Unfortunately, collecting large datasets with microbiome and genotype data is challenging. Currently, the largest PD datasets that have both genotype and microbiome data are the two datasets used here, one has 199 PD and 117 controls and the other 312 PD and 174 controls. A major challenge is to secure well-coordinated studies with large sample sizes that can be pooled or meta-analyzed. Unlike genetic studies which can be combined thanks to the stability of DNA, combining microbiome studies is challenging due to the effects of collection and storage parameters on outcomes. Standardization of methods can alleviate some of the cross-study variations. It is also more difficult to collect stool samples than blood or even saliva. People are averse to donating stool samples; 30% of our research participants who donated blood refused to donate stool. Microbiome researchers are cognizant of the need to join resources, create standardized protocols, and coordinate data collection across laboratories. Within a few years, we will be able to amass the sample sizes needed to address the interaction of genes, environment, and microbiome on a comprehensive scale. Here, limited by sample size, we chose to explore one PD-associated locus (*SNCA*) and three PD-associated opportunistic pathogens, hoping that the resulting data will help formulate testable hypotheses.

Our rationale for choosing *SNCA* and opportunistic pathogen as our candidate gene and candidate taxa stemmed from the collective literature. *SNCA* is a key player in PD. Alpha-synuclein aggregates are a pathologic hallmark of PD. Mutations in *SNCA* cause autosomal dominant PD and variants that affect *SNCA* gene expression are the most significant genetic risk factors for idiopathic PD^23,32^. While the functions of alpha-synuclein are yet to be fully understood, it has been shown to play a key role in activating the immune system, acting as antigen presented by PD-associated major histocompatibility molecules and recognized by T cells which infiltrate the brain^33–35^. *SNCA* expression has also been shown to be critical for inducing immune response against infections unrelated to PD^27,28^. Alpha-synuclein aggregates, which have historically been considered as a marker of PD pathology in the brain, can actually form in the enteric neurons^19^ and in animal models have been shown to propagate from the gut to the brain^22^ possibly via the vagus nerve^36,37^. The trigger that induces alpha-synuclein pathology in the gut is unknown. Braak hypothesized the trigger is a pathogen^17,18^. Our choice of opportunistic pathogens as the candidate taxa for interaction testing was driven by our recent finding of an overabundance of opportunistic pathogens in PD gut and Braak’s hypothesis. Moreover, a study conducted in mice has corroborated that intestinal infection triggers dopaminergic cell loss and motor impairment in a *Pink1* knockout model of PD^38^. Whether the opportunistic pathogens found in human PD microbiome are triggers of PD is being investigated. In the meantime, we thought that if these opportunistic pathogens are involved in PD pathogenesis, there is likely a connection to *SNCA* genotype worth exploring.

Interestingly, three different *SNCA*-linked genetic variants emerged as potential modifiers for the association of the three opportunistic pathogens with PD. They are independent of each other with no LD among them. All three interacting variants are eQTLs for *SNCA* and lncRNAs that affect the expression of *SNCA*. lncRNA are emerging as important regulators of gene expression^39^. Aberrant expression of lncRNA has been widely reported in PD, often in relation to the expression and aggregation of *SNCA*^40,41^. More specific to the present findings, the lncRNA’s near *SNCA*, including *SNCA-AS1* identified here, were shown to be under-expressed while *SNCA* mRNA was over-expressed in substantia nigra of autopsied PD brains compared to controls^30^. Another lncRNA identified here, *RP11-115D19.1*, was shown to repress *SNCA* expression in SH-SY5Y human neuroblastoma cell lines^29^. This suggests a link between *SNCA* expression and the presence of opportunistic pathogens, and that regulation of this link may involve different regulatory elements depending on the pathogen. lncRNA is expressed in a cell-specific manner^42^. It is not known which cells in the gut are responsible for the expression and corruption of alpha-synuclein into pathologic species. If the opportunistic pathogens induce *SNCA* expression or corruption, they may do so by signaling different cell types, hence the involvement of different regulatory elements. *Prevotella* and *Porphyromonas* are commensal to gastrointestinal and urinary track, *Corynebacterium* is common in skin microbiome. All three can be found at low abundance in the gut. All three have been implicated in causing infections in nearly every type of tissue (reviewed by Wallen et al.^11^).

These data provide new leads and hypotheses that with follow-up in experimental models may yield a better understanding of disease pathogenesis. These data alone cannot resolve cause and effect. We cannot tell if the *SNCA* genotype leads to altered colonization of the gut, which in turn leads to PD, or is it the other way around, *SNCA* genotype causes PD, which leads to gut dysfunction and accumulation of pathogens. Or, maybe the pathogen induces alpha-synuclein expression which elicits immune response to infection as seen in other infections unrelated to PD, but in individuals with certain regulatory genotypes at *SNCA*, the alpha-synuclein expression goes into overdrive and PD is a downstream consequence. An alternative hypothesis for the interaction of *SNCA* eQTL and an opportunistic pathogen is that eQTL controls alpha-synuclein concentration in the cell, bacteria triggers misfolding and aggregation of alpha-synuclein, and since misfolding and aggregation is directly dependent on the concentration of alpha-synuclein in the cell^43^, individuals with certain *SNCA* eQTL genotypes are at higher risk of developing PD pathology from gut-derived insults. One can further speculate that these bacteria might promote alpha-synuclein misfolding and aggregation by invading the host cells (all three can invade host cells) or via producing toxic or proinflammatory substances. *Prevotella* and *Porphyromonas* produce lipopolysaccharides, gut-derived proinflammatory endotoxins that when administered to mice, cause intestinal permeability and progressive increase in alpha-synuclein expression in the gut, and neuroinflammation and nigral neurodegeneration in the brain^44,45^. Further studies in humans conducted over time and in experimental models will be needed to tease out the underlying biology of these interactions.

In conclusion, this study was exploratory and hypothesis generating. Within this cautionary framework, this study suggests that genetic susceptibility to disease and the dysbiosis in the gut microbiome are not operating independently. Rather, it suggests that alterations in gut microbiome should be integrated in the gene–environment interaction paradigm, which has long been suspected to be the cause of idiopathic disease but is yet to produce a causative combination. The results also put forth the hypothesis that the PD-associated genetic variants may confer susceptibility via interaction with microbiome; opening a new area to search for the incomplete penetrance of PD susceptibility genes. In addition, while it is yet to be seen if the opportunistic pathogens are part of the cause or consequence of disease (experiments are underway), the finding that their abundance correlated with PD-associated genotypes adds credence to the hypotheses that their presence signifies a role in disease pathogenesis, possibly as the triggers that Braak originally proposed. With the identity of the candidate microorganisms in hand, these hypotheses can be tested in model systems. Thus, the significance of this work lies not on achieving conclusive discoveries, rather on generating novel hypotheses with tangible leads that can be put to test experimentally.

## Methods

### Subjects

The study was approved by the institutional review boards at all participating institutions, namely New York State Department of Health, University of Alabama at Birmingham, VA Puget Sound Health Care System, Emory University, and Albany Medical Center. All subjects provided written informed consent for their participation. This study included two datasets each composed of persons with PD (case) and neurologically healthy individuals (control). Subject enrollment and data collection for both datasets were conducted by the NeuroGenetics Research Consortium (NGRC) team using uniform protocols. The two datasets used here were the same datasets used by Wallen et al for characterizing the microbiome^11^; except here we have generated and added genetic data, and subjects without genotype were excluded (Supplementary Table 1). Methods of subject selection and data collection have been described in detail before^11^. Briefly, PD was diagnosed by NGRC-affiliated movement disorder specialists^46^. Controls were self-reported free of neurological disease. Metadata were collected on over 40 variables including age, sex, race, geography, diet, medication, health, gastrointestinal issues, weight fluctuation, and body mass index. We enrolled 212 persons with PD and 136 controls in 2014 (dataset 1)^47^, and 323 PD and 184 controls during 2015–2017 (dataset 2)^11^. Subsequently, we excluded 11 PD and 4 control samples for failing 16S sequencing, 2 PD for unreliable metadata, and 15 controls for lacking genotypes from dataset 1; and 11 PD and 10 controls were excluded from dataset 2 for lacking genotype data. The sample size used in current analyses was 199 PD and 117 controls in dataset 1, and 312 PD and 174 controls in dataset 2 (Supplementary Table 1).

### Microbiome data

Methods for collection, processing, and analysis of microbiome data have been reported in detail^11^, and raw sequences are publicly available at NCBI SRA BioProject ID PRJNA601994. Each subject provided a single stool sample at a single time point, and each sample was measured once. Briefly, for both datasets uniformly, DNA/RNA-free sterile cotton swabs were used to collect stool, DNA was extracted using MoBio extraction kits, and 16S rRNA gene hypervariable region 4 was sequenced using the same primers, but in two laboratories, resulting in 10x greater sequencing depth in dataset 2 than dataset 1. Sequences were demultiplexed using QIIME2 (core distribution 2018.6)^48^ for dataset 1 and BCL2FASTQ (Illumina, San Diego, CA) for dataset 2. Bioinformatics processing of sequences was performed separately for each dataset, but using an identical pipeline (see Wallen et al.^11^ for step-by-step protocol). Unique amplicon sequence variants (ASVs) were identified using DADA2 v 1.8^49^ and given taxonomic assignment using DADA2 and SILVA (v 132) reference database. Analyses were performed at genus/subgenus/clade level (here, referred to as taxa). Taxa that were associated with PD were then investigated at species level. This was important because not all species of *Corynebacterium_1*, *Porphyromonas*, and *Prevotella* are opportunistic pathogens. Species that made up each taxon were identified by SILVA when an ASV matched a species at 100% homology. To augment SILVA, we blasted ASVs that made up *Corynebacterium_1*, *Porphyromonas*, and *Prevotella* against the NCBI 16S rRNA database for matches that were >99–100% identical with high statistical confidence.

### Defining *SNCA* region

Since the expression of *SNCA* has been implicated in PD and the most significant genetic markers of PD map outside *SNCA* and are eQTL for *SNCA*, we set out to explore the entire region that includes known *cis-*eQTLs for *SNCA*. We used GTEx (V8 release) database and searched for eQTLs for *SNCA* (https://gtexportal.org/home/gene/SNCA). The search returned 1,749 entries which included 601 unique eQTLs. They span from ch4:90.6 Mb at 5′ upstream *SNCA* to ch4:88.9 Mb at 3′ downstream *SNCA* (GRCh38/hg38). We had genotypes for 2,627 SNPs in this region (excluding SNPs with MAF < 0.1 and imputation quality score <0.8), and among them, we had captured 413 of the 601 eQTLs for *SNCA*. Interaction test was conducted for all 2,627 SNPs and the SNP with the highest interaction *P* value was chosen for genotype-stratified analysis.

### Genotype data

Genotype data for the *SNCA* region were extracted from GWAS data. Since only some of the GWAS data have been published and most were generated recently and unpublished, we will provide the methods in detail. Dataset 1 is composed of a subset of the NGRC subjects who were genotyped in 2009 using Illumina HumanOmni1-Quad array (GWAS published in 2010)^35^ and were subsequently enrolled for microbiome study, and additional NGRC samples that were collected for microbiome studies in 2014 who were genotyped in 2018 using Illumina Infinium Multi-Ethnic array (unpublished data). Dataset 2 was enrolled into NGRC in 2015–2017 and genotyped in 2020 using Infinium Global Diversity Array (unpublished data). Genotyping and quality control (QC) of SNP genotypes are described below. Unless otherwise specified, QC was performed using PLINK 1.9 (v1.90b6.16)^50^.

Approximately 70% of subjects in dataset 1 (*N* = 244) were genotyped in 2009 using the HumanOmni1-Quad_v1-0_B BeadChip for a GWAS of PD^35^, resulting in genotypes for 1,012,895 SNPs. Subjects were also genotyped using the Illumina Immunochip resulting in genotypes for 202,798 SNPs. QC of genotype data had been previously performed using PLINK v1.07^35^, therefore, this process was redone for current study using an updated version of PLINK v1.9. The mean non-Y chromosome call rate for samples in both arrays was 99.9%. Calculation of identity-by-descent in PLINK using HumanOmni genotypes revealed no cryptic relatedness between samples (PI_HAT >0.15). A subset of SNP mappings were in NCBI36/hg18 build, and were converted to GRCh37/hg19 using the liftOver executable and hg18ToHg19.over.chain.gz chain file from UCSC genome browser (downloaded from https://hgdownload.soe.ucsc.edu/downloads.html). SNP filtering for both HumanOmni and Immunochip genotypes included removal of SNPs with call rate <99%, Hardy-Weinberg equilibrium (HWE) *P* value < 1E−6, MAF <0.01, and MAF difference between sexes >0.15. HumanOmni and Immunochip data were then merged, and SNPs with significant differences in PD patient and control missing rates (*P* < 1E−5) and duplicate SNPs were removed. To remove duplicate SNPs, we first checked the genotype concordance between duplicated SNPs. If duplicate SNPs were concordant, we took the SNP with the lowest missing rate, or the first listed SNP if missing rates were the same. If duplicate SNPs were discordant, we removed both SNPs as we do not know which SNP is correct. After QC, the remaining number of genotyped SNPs was 910,083 with a mean call rate of 99.8%.

Approximately 30% of subjects in dataset 1 (*N* = 89) were enrolled after the 2010 PD GWAS. These samples were genotyped in 2018 using the Infinium Multi-Ethnic EUR/EAS/SAS-8 array. Raw genotyping intensity files were uploaded to GenomeStudio v 2.0.4 where genotype cluster definitions and calls were determined for each SNP using intensity data from all samples. The GenCall (genotype quality score) threshold for calling SNP genotypes was set at 0.15, and SNPs that resulted in a genotype cluster separation <0.2 were zeroed out for their genotype. Genotypes for 1,649,668 SNPs were then exported from GenomeStudio using the PLINK plugin v 2.1.4, and converted to PLINK binary files for further QC. The mean non-Y chromosome call rate for samples was 99.8%. Calculation of identity-by-descent revealed no cryptic relatedness among samples (PI_HAT <0.15). A subset of SNP mappings were in GRCh38/hg38 build, and were converted to GRCh37/hg19 using the liftOver executable and hg38ToHg19.over.chain.gz chain file. The same SNP filtering criteria were implemented here as described above for the first group in dataset 1: call rate <99%, HWE *P* value < 1E−6, MAF <0.01, MAF difference between sexes >0.15, significant differences in PD patient and control missing rates (*P* < 1E−5), and removal of duplicate SNPs. After QC, the remaining number of genotyped SNPs was 749,362 with a mean call rate of 100%.

All subjects in dataset 2 (*N* = 486) were genotyped at once in 2020 using the Infinium Global Diversity Array. Genotype clusters were defined using GenomeStudio v 2011.1 and 99% of the genotyped samples. Genotypes were not called for SNPs with GenCall score <0.15, and failure criteria for autosomal and X chromosome SNPs included the following: call rate <85%, MAF ≤ 1% and call rate <95%, heterozygote rate ≥80%, cluster separation <0.2, any positive control replicate errors, absolute difference in call rate between genders >10% (autosomal only), absolute difference in heterozygote rate between genders >30% (autosomal only), and male heterozygote rate greater than 1% (X only). All Y chromosome, XY pseudo-autosomal region (PAR), and mitochondrial SNPs were manually reviewed. Genotypes for 1,827,062 SNPs were released in the form of PLINK binary files. The mean non-Y chromosome call rate for samples was 99.2%. Calculation of identity-by-descent showed two subjects were genetically related as a parent and offspring (PI_HAT = 0.5), which we were already aware of. The same SNP filtering criteria was implemented here as it was for dataset 1: call rate <99%, HWE *P* value < 1E−6, MAF <0.01, MAF difference between sexes >0.15, significant differences in PD patient and control missing rates (*P* < 1E−5), and removal of duplicate SNPs. After QC, the remaining number of SNPs for dataset 2 was 783,263 with a mean call rate of 99.9%.

### Principal component analysis (PCA)

We performed PCA for each genotyping array using 1000 Genomes Phase 3 reference genotypes. Study genotypes were first merged with 1000 Genomes Phase 3 genotypes (previously filtered for non-triallelic SNPs and SNPs with MAF >5%) using GenotypeHarmonizer v 1.4.23^51^ and PLINK. Merged genotypes were then LD-pruned as previously described^35^, resulting in a mean LD-pruned subset of ~148,000 SNPs. Principal components were calculated using pruned SNPs and the top two PCs were plotted using ggplot2 (Supplementary Fig. 1).

### Imputation

To increase SNP density, we imputed genotypes using Minimac4^52^ on Trans-Omics for Precision Medicine (TOPMed) Imputation Server (https://imputation.biodatacatalyst.nhlbi.nih.gov)^53^. To be compatible with TOPMed, we converted SNP coordinates to GRCh38/hg38 using the liftOver executable and hg19ToHg38.over.chain.gz chain file. SNP mappings were then checked and corrected for use with TOPMed reference panels using the utility scripts HRC-1000G-check-bim.pl (v4.3.0) and CreateTOPMed.pl (downloaded from https://www.well.ox.ac.uk/~wrayner/tools/), and a TOPMed reference file ALL.TOPMed_freeze5_hg38_dbSNP.vcf.gz (downloaded from https://bravo.sph.umich.edu/freeze5/hg38/download). Running of these utility scripts resulted in a series of PLINK commands to correct genotypes files for concordance with TOPMed by excluding SNPs that did not have a match in TOPMed, mitochondrial SNPs, palindromic SNPs with frequency >0.4, SNPs with non-matching alleles to TOPMed, indels, and duplicates. Once running of PLINK commands was complete, genotype files were converted to variant call format (VCF) and submitted to the TOPMed Imputation Server using the following parameters: reference panel TOPMed version r2 2020, array build GRCh38/hg38, *r*^2^ filter threshold 0.3 (although we excluded from downstream analyses SNPs with *r*^2^ <0.8), Eagle v2.4 for phasing, skip QC frequency check, and run in QC & imputation mode. VCF files with genotypes and imputed dosage data were then outputted by the imputation server and used in statistical analyses. Directly genotyped and imputed genotypes from HumanOmni1-Quad_v1-0_B BeadChip and Infinium Multi-Ethnic EUR/EAS/SAS-8 Kit arrays were merged to create dataset 1. To merge genotypes, one duplicate subject was first removed from the Infinium Multi-Ethnic array VCF files. Then, per chromosome VCF files were merged by first indexing the files using tabix, then merging the files using bcftools’ merge function (tabix and bcftools v 1.10.2). The genome-wide data included 20,263,129 SNPs (1,282,026 genotyped and 18,981,103 imputed) for dataset 1 and 21,389,007 SNPs (719,329 genotyped and 20,669,678 imputed) for dataset 2.

For the present study, the *SNCA* region was defined as ch4:88.9Mb-90.6 Mb (as described above). SNPs within *SNCA* region with MAF<0.1 were excluded as there would be too few homozygotes for stratified analysis. Imputed SNPs with imputation quality score *r*^2^ <0.8 were also excluded. Analysis included 2,627 SNPs that were directly genotyped or imputed in both datasets.

### Statistical analysis overview

For all analyses, raw taxa abundances were transformed using the centered log-ratio (clr) transformation before including in tests. The clr transformation was performed using Eq. (1) in R:

1 $$\left[ {{{{\mathrm{clr}}}}\left( {{{{\mathrm{X}}}}_{{{{\mathrm{taxa}}}}}} \right)={{{\mathrm{log}}}}\left( {{{{\mathrm{X}}}}_{{{{\mathrm{taxa}}}}}} \right) - {{{\mathrm{mean}}}}\left( {{{{\mathrm{log}}}}\left( {{\it{X}}_{\it{1}}{{{\mathrm{,}}}}{\it{X}}_{\it{2}} \ldots {\it{X}}_{\it{n}}} \right)} \right)} \right]$$

where *X*_taxa_ is the raw abundance of either *Corynebacterium_1*, *Porphyromonas*, or *Prevotella* in a single sample with a pseudocount of 1 added, and *X*_*1*_*,X*_*2*_*…X*_*n*_ are the raw abundances of every taxon detected in the same sample with a pseudocount of 1 added.

Throughout, tests were conducted in two datasets separately, and results were meta-analyzed using fixed- and random-effect models, and tested for heterogeneity. If heterogeneity was detected across two datasets (Cochran’s Q *P* < 0.1), random-effect meta-analysis results were reported. If no heterogeneity was detected (Cochran’s Q *P* ≥ 0.1), fixed-effect results were reported. *P* values were all two-tailed.

### Screening for interaction

We tested interaction to identify candidate SNPs that may modify the association of *Corynebacterium_1*, *Porphyromonas*, or *Prevotella* with PD. For each dataset separately, linear regression was performed using PLINK 2 (v2.3 alpha) --glm function to test the interaction between case/control status and SNP on the abundance of each taxon. Equation (2) shows the model that was specified for the analyses:

2 $$\left[ {{{{\mathrm{Taxon}}}}\sim \left( {{{{\mathrm{SNP}}}}\;{{{\mathrm{x}}}}\;{{{\mathrm{case}}}}/{{{\mathrm{control}}}}} \right) + {{{\mathrm{SNP}}}} + {{{\mathrm{case}}}}/{{{\mathrm{control}}}} + {{{\mathrm{sex}}}} + {{{\mathrm{age}}}}} \right]$$

where taxon is the clr-transformed abundance of *Corynebacterium_1*, *Porphyromonas*, or *Prevotella*, and SNP is genotype defined as dosages of the minor allele ranging from 0 to 2 in the additive model. The interaction test was adjusted for sex, age, and main effects of case/control status and SNP. Interaction β and standard errors generated for each taxon were then used as input for meta-analysis in METASOFT v2.0.1^54^. Summary statistics are in Supplementary Tables 2–4. For each taxon, the SNP that reached the highest statistical significance in meta-analysis was tagged as candidate interacting SNP.

### Linkage disequilibrium

To visualize the results across the *SNCA* region, results from meta-analyses were uploaded to LocusZoom^55^. LD between SNPs was calculated in LocusZoom based on the “EUR” LD population. The resulting plots show the location of the SNPs tested in the region and their LD with candidate interacting SNP (Fig. 1a–c).

To determine if the three candidate interacting SNPs were correlated, possibly tagging the same variant, or independent, pairwise LD estimates were calculated using the LDpair tool with 1000 Genome phase 3 European data from LDlink v4.1^56^.

### Association of taxa with PD as a function of genotype

Subjects were grouped by their genotype at the interacting SNP. We used the best guessed genotype for the imputed SNPs and directly genotyped SNPs. Association of each taxon with PD (case/control status) was tested within each genotype, while adjusting for age and sex, using linear regression via the R function glm from the stats v 3.5.0 package. Odds ratios (OR) and corresponding *P* values were calculated using linear regression. Each dataset was analyzed separately. Meta-analysis was performed using the metagen function of the meta R package v4.9.7, specifying the summary measure to be “OR”. Results are shown in Table 1. Boxplots were created using ggplot2 v 3.1.0 (Fig. 2). Of the two variants of each SNP, the one that was associated with enhanced differential abundance in PD vs. controls was tagged as the effect allele.

### Association of interacting SNP with PD

To test whether the interacting SNP had a main effect on PD risk, we used Firth’s penalized logistic regression (logistf R package v 1.23) testing SNP genotype (dosages of the effect allele ranging from 0 to 2) in an additive model against case-control status adjusting for age and sex. OR, SE and *P* values were calculated. Results were meta-analyzed using a fixed-effects model as implemented in the metagen function, of the meta R package v4.9.7, specifying the summary measure to be “OR”.

### Functional analysis in silico

While we had defined the S*NCA* region such that it encompassed known eQTLs, only 413 of 2,676 SNPs tested were eQTL. Thus, if left to chance, the odds that a candidate SNP would be an eQTL was ~15%. We used UCSC Genome Browser (hg38 build) to map the candidate SNPs and visually inspect if they were in a regulatory sequence. To determine, for each SNP, if they were found in genome-wide studies to be significantly associated with gene expression, we used two eQTL databases, GTEx (https://gtexportal.org) and eQTLGen (https://www.eqtlgen.org).

### Reporting summary

Further information on research design is available in the Nature Research Reporting Summary linked to this article.

## Supplementary information

Supplementary Data

Reporting Summary

## Data Availability

All data that are necessary to generate, verify and extend the research in the article are publicly available. Individual-level raw 16S sequences and basic metadata are publicly available at NCBI Sequence Read Archive (SRA) BioProject ID PRJNA601994. Summary statistics of interaction of 2,627 SNPs in *SNCA* region with PD on clr-transformed abundances of taxa are provided in Supplementary Table 2 for *Coryenbacterium_1*, Supplementary Table 3 for *Porphyromonas*, and Supplementary Table 4 for *Prevotella*. Individual-level SNP (2,627 SNPs) and phenotype data (sex, age, case/control) that were used in this paper (the *SNCA* region) are provided in Supplementary Table 5. The full genome-wide genotypes and phenotype (not used in this article) will be available on dbGaP (accession code phs000196) one year from publication of this article to allow authors time to analyze the data.

## Code availability

No custom codes were used. All software and packages, their versions, relevant specification and parameters are stated in the Methods section.
