# Supplementary material for: Exploring human-genome gut-microbiome interaction in Parkinson’s disease
Source: NPJ Parkinsons Dis. 2021 Aug 18;7:74. doi: 10.1038/s41531-021-00218-2 (PMC8373869; doi:10.1038/s41531-021-00218-2)
Supplement: Supplementary file 2 — Reporting Summary [file 41531_2021_218_MOESM2_ESM.pdf]

nature research

Corresponding author(s): Haydeh Payami

Last updated by author(s): Jun 4, 2021

Reporting Summary

Nature Research wishes to improve the reproducibility of the work that we publish. This form provides structure for consistency and transparency in reporting. For further information on Nature Research policies, see our Editorial Policies and the Editorial Policy Checklist.

Statistics

For all statistical analyses, confirm that the following items are present in the figure legend, table legend, main text, or Methods section.

n/a	Confirmed	
☐	☒	The exact sample size (n) for each experimental group/condition, given as a discrete number and unit of measurement
☐	☒	A statement on whether measurements were taken from distinct samples or whether the same sample was measured repeatedly
☐	☒	The statistical test(s) used AND whether they are one- or two-sided<br>*Only common tests should be described solely by name; describe more complex techniques in the Methods section.*
☐	☒	A description of all covariates tested
☐	☒	A description of any assumptions or corrections, such as tests of normality and adjustment for multiple comparisons
☐	☒	A full description of the statistical parameters including central tendency (e.g. means) or other basic estimates (e.g. regression coefficient) AND variation (e.g. standard deviation) or associated estimates of uncertainty (e.g. confidence intervals)
☐	☒	For null hypothesis testing, the test statistic (e.g. F, t, r) with confidence intervals, effect sizes, degrees of freedom and P value noted<br>*Give P values as exact values whenever suitable.*
☒	☐	For Bayesian analysis, information on the choice of priors and Markov chain Monte Carlo settings
☒	☐	For hierarchical and complex designs, identification of the appropriate level for tests and full reporting of outcomes
☐	☒	Estimates of effect sizes (e.g. Cohen's d, Pearson's r), indicating how they were calculated

Our web collection on statistics for biologists contains articles on many of the points above.

Software and code

Policy information about availability of computer code

Data collection	No software used
Data analysis	R v 3.5.0; QIIME2 (core distribution 2018.6); cutadapt v 1.16; DADA2 R package v 1.8; phyloseq R package v 1.24.2; ggplot2 R package v 3.1.0; PLINK v1.9b6.16; GenomeStudio v 2.0.4; PLINK plugin for GenomeStudio v 2.1.4; GenotypeHarmonizer v 1.4.23; HRC-1000G-check-bim.pl v 4.3.0; tabix and bcftools v 1.10.2; PLINK 2 v 2.3 alpha; METASOFT v 2.0.1; LocusZoom v0.13.3; LDlink v 4.1; meta v 4.9.7; logistf v 1.23

For manuscripts utilizing custom algorithms or software that are central to the research but not yet described in published literature, software must be made available to editors and reviewers. We strongly encourage code deposition in a community repository (e.g. GitHub). See the Nature Research guidelines for submitting code & software for further information.

Data

Policy information about availability of data

All manuscripts must include a data availability statement. This statement should provide the following information, where applicable:

- Accession codes, unique identifiers, or web links for publicly available datasets
- A list of figures that have associated raw data
- A description of any restrictions on data availability

Individual-level raw 16S sequences and basic metadata are publicly available at NCBI Sequence Read Archive (SRA) BioProject ID PRJNA601994. Genetic data and summary statistics of interaction of 2,627 SNPs in SNCA region with PD on clr transformed abundances of taxa are provided in Supplementary Table 2 for Coryenbacterium_1, Supplementary Table 3 for Porphyromonas, and Supplementary Table 4 for Prevotella.

Field-specific reporting

Please select the one below that is the best fit for your research. If you are not sure, read the appropriate sections before making your selection.

☒ Life sciences ☐ Behavioural & social sciences ☐ Ecological, evolutionary & environmental sciences

For a reference copy of the document with all sections, see nature.com/documents/nr-reporting-summary-flat.pdf

Life sciences study design

All studies must disclose on these points even when the disclosure is negative.

Sample size	Dataset 1 sample size (N=316) was determined by the time constraint on available funding (less than one year in 2014) and the availability of genotype data after genotyping; we collected as many samples as possible at the three NeuroGenetics Research Consortium (NGRC) affiliated movement disorder clinics in the given time period and submitted as many high quality DNA samples as possible for genotyping. Dataset 2 sample size (N=486) reflects the number of individuals that could be enrolled in a three year period (2015-2017) from a fourth NGRC-affiliated movement disorder clinic and available genotype data after genotyping. Consent and compliance rate was markedly lower for donation of stool than blood.
Data exclusions	15 subjects from dataset 1 whose stool samples did not yield any sequence or yielded too few sequences to be analyzed were excluded from all analyses (these samples have already been excluded from the publicly available data). 15 subjects from dataset 1 were excluded from analyses due to no available genotype data. 21 subjects from dataset 2 were excluded from analyses due to no available genotype data. Two subjects from dataset 1 whose metadata were deemed unreliable were excluded from analyses (sample IDs on publicly available data: 10122.FP0016201 and 10122.GMWA.1090).
Replication	Two independent datasets were used. Analyses were conducted in each dataset separately, then results combined using meta-analysis. If heterogeneity existed between datasets for any analyses, a random-effects model results were reported to account for the heterogeneity.
Randomization	Case and control samples were randomized on plates for sequencing and genotyping to avoid batch effect.
Blinding	Investigators conducting genotyping and 16S sequencing were blinded to case-control status.

Reporting for specific materials, systems and methods

We require information from authors about some types of materials, experimental systems and methods used in many studies. Here, indicate whether each material, system or method listed is relevant to your study. If you are not sure if a list item applies to your research, read the appropriate section before selecting a response.

Materials & experimental systems

n/a	Involved in the study	
☒	☐	Antibodies
☒	☐	Eukaryotic cell lines
☒	☐	Palaeontology and archaeology
☒	☐	Animals and other organisms
☐	☒	Human research participants
☒	☐	Clinical data
☒	☐	Dual use research of concern

Methods

n/a	Involved in the study	
☒	☐	ChIP-seq
☒	☐	Flow cytometry
☒	☐	MRI-based neuroimaging

Human research participants

Policy information about studies involving human research participants

Population characteristics	Subjects included persons with Parkinson's disease and controls, of both genders, ages 40 years old or older. Population characteristics relevant to analyses performed in this study are described in the manuscript.
Recruitment	Participants were recruited in four states in the US, as described in the manuscript.
Ethics oversight	The study was approved by institutional review boards for ethical conduct of human subject research at all participating institutions; namely, New York State Department of Health, University of Alabama at Birmingham, VA Puget Sound Health Care System, Emory University, and Albany Medical Center.

Note that full information on the approval of the study protocol must also be provided in the manuscript.
